# Recombinant Zoster Vaccination Among US Veterans Receiving Immunosuppressive Medications

**DOI:** 10.1001/jamanetworkopen.2024.39945

**Published:** 2024-10-11

**Authors:** Sharon Abada, Jing Li, Gary Tarasovsky, Cherish Wilson, Jinoos Yazdany, Mary A. Whooley, Gabriela Schmajuk

**Affiliations:** 1 University of California San Francisco; 2 San Francisco VA Medical Center, San Francisco, California; 3 Institute for Health Policy, University of California San Francisco

## Abstract

This cross-sectional study investigates rates of recombinant zoster vaccination among US veterans receiving immunosuppressive medications before and after expanded indications for younger adults who are immunocompromised.

## Introduction

Individuals who are immunocompromised, including those receiving immunosuppressive medications, have an increased risk of herpes zoster and associated complications compared with the general population; those receiving targeted synthetic disease modifying agents (tsDMARDs), such as Janus kinase inhibitors, are at particularly increased risk.^1,2^ In 2017, the Food and Drug Administration (FDA) approved a recombinant zoster vaccine (RZV) for individuals aged 50 years or older. In 2021, the FDA additionally approved RZV for adults aged 18 years and older who are immunocompromised, and the Centers for Disease Control and Prevention and American College of Rheumatology issued updated guidance in 2021 and 2022, respectively.^3,4,5^

However, there is little published evidence regarding RZV vaccination among US adults who are immunocompromised, particularly those younger than 50 years. We assessed RZV receipt among veterans receiving immunosuppressive medications within the Veterans Health Administration (VHA) health care system before and after expanded indications.

## Methods

This cross-sectional study was approved by the VA Quality Enhancement Research Initiative and University of California San Francisco institutional review board with a waiver of informed consent as a minimal-risk study. It used nationwide VHA data among veterans at 130 medical facilities prescribed 1 or more immunosuppressive medications for 90 days or more between January 1, 2018, and June 30, 2023. The outcome was the percentage of veterans with 1 or more RZV doses documented during the study period, assessed before the expanded indication (January 2018 to February 2022) and at any time (January 2018 to June 2023). To examine factors associated with RZV receipt at any time, we constructed a multilevel regression model, including age, sex, self-identified race and ethnicity, rurality of residence, visits with more than 1 subspecialty (yes or no), receipt of 1 or more other vaccination at the VA (yes or no), and immunosuppressive medication type, accounting for clustering by facility using generalized estimating equations (eMethods in Supplement 1). Facility-level performance was reported as the percentage of veterans with 1 or more RZV doses documented among those eligible at any time. Analyses were performed using Stata statistical software version 16 (StataCorp) (eMethods in Supplement 1). We followed the STROBE reporting guideline.

## Results

We identified 190 162 veterans receiving immunosuppressive medications across 130 facilities during the study period. By the end of the study period, 23 295 veterans (12.3%) were younger than 50 years (characteristics shown in Table 1).

**Table 1.** Demographic Characteristics of the Study Sample

Characteristic	Veterans, No. (%)			*P* value^a^
	Total (N = 190 162)	Any RZV (n = 86 235)	No RZV (n = 103 927)	
Age, y				
Mean (SD)	67.8 (13.8)	70.6 (10.6)	65.5 (15.5)	<.001
<50	23 295 (12.3)	3118 (3.6)	20 177 (19.4)	
≥50	166 867 (87.7)	83 117 (96.4)	83 750 (80.6)	
Sex				
Female	23 167 (12.2)	9655 (11.2)	13 512 (13.0)	<.001
Male	166 995 (87.8)	76 580 (88.8)	90 415 (87.0)	
Self-identified race				
African American	32 609 (17.1)	13 323 (15.4)	19 286 (18.6)	<.001
Asian	1936 (1)	907 (1.1)	1029 (1.0)	
White	139 744 (73.5)	65 066 (75.5)	74 678 (71.9)	
Other^b^	3485 (1.8)	1546 (1.8)	1939 (1.9)	
Unknown	12 388 (6.5)	5393 (6.3)	6995 (6.7)	
Self-identified ethnicity				
Hispanic	10 964 (5.8)	4733 (5.5)	6231 (6.0)	<.001
Not Hispanic	171 025 (89.9)	77 937 (90.4)	93 088 (89.6)	
Unknown	8173 (4.3)	3565 (4.1)	4608 (4.4)	
Facility region				
Continental	33 352 (17.5)	13 491 (15.6)	19 861 (19.1)	<.001
Midwest	46 064 (24.2)	23 633 (27.4)	22 431 (21.6)	
North Atlantic	42 318 (22.3)	19 983 (23.2)	22 335 (21.5)	
Pacific	31 633 (16.6)	14 769 (17.1)	16 864 (16.2)	
Southeast	36 795 (19.3)	14 359 (16.7)	22 436 (21.6)	
Facility rurality				
Urban	119 698 (62.9)	54 277 (62.9)	65 421 (62.9)	<.001
Rural	69 843 (36.7)	31 789 (36.9)	38 054 (36.6)	
Unknown	621 (0.3)	169 (0.2)	452 (0.4)	
Rheumatic disease^c^				
Rheumatoid arthritis	52 665 (27.7)	26 334 (30.5)	26 331 (25.3)	<.001
Other inflammatory arthritis	9402 (4.9)	5066 (5.9)	4336 (4.2)	<.001
Spondylitis	8037 (4.2)	3467 (4.0)	4570 (4.4)	<.001
Systemic lupus erythematosus	7626 (4.0)	3088 (3.6)	4538 (4.4)	<.001
Sarcoidosis	4449 (2.3)	2005 (2.3)	2444 (2.4)	.70
ANCA-associated vasculitis	902 (0.5)	426 (0.5)	476 (0.5)	.26
MCTD	763 (0.4)	327 (0.4)	436 (0.4)	.17
Other	19 867 (10.4)	10 454 (12.1)	9413 (9.1)	<.001
Immunosuppressive medication				
csDMARD monotherapy	71 822 (37.8)	32 388 (37.6)	39 434 (37.9)	<.001
GC monotherapy	37 783 (19.9)	17 783 (20.6)	20 000 (19.2)	
Biologic monotherapy	36 043 (19.0)	14 210 (16.5)	21 833 (21.0)	
Combination of csDMARD, GC, or biologic (no tsDMARDs)	35 995 (18.9)	17 343 (20.1)	18 652 (17.9)	
tsDMARD monotherapy or in combination	8519 (4.5)	4511 (5.2)	4008 (3.9)	
Other vaccination since 2018	178 250 (93.7)	85 660 (99.3)	92 590 (89.1)	<.001
≥1 Subspecialty clinic visit since 2018				
Rheumatology	89 221 (46.9)	43 740 (50.7)	45 481 (43.8)	<.001
Gastroenterology	72 582 (38.2)	35 926 (41.7)	36 656 (35.3)	<.001
Neurology	47 592 (25.0)	23 774 (27.6)	23 818 (22.9)	<.001
Oncology	33 716 (17.7)	15 342 (17.8)	18 374 (17.7)	.53
Visits with >1 specialty since 2018	71 804 (37.8)	36 268 (42.1)	35 536 (34.2)	<.001
No specialty visits since 2018	44 359 (23.3)	17 511 (20.3)	26 848 (25.8)	<.001

Abbreviations: ANCA, antineutrophil cytoplasmic antibody; csDMARD, conventional synthetic disease-modifying antirheumatic drug; GC, glucocorticoid; MCTD, mixed connective tissue disease; tsDMARD, targeted synthetic disease-modifying antirheumatic drug.

^a^ *P* values were tested by the *t* test for continuous variables and χ^2^ test for categorical variables.

^b^ Other race includes American Indian or Alaska Native and Native Hawaiian or Other Pacific Islander.

^c^ Rheumatic disease diagnoses are not mutually exclusive.

Among veterans aged 50 years or older, 55 546 of 153 620 individuals (36.2%) had an RZV before the expanded indication and 83 117 of 166 867 individuals (49.8%) had it by mid-2023. Among veterans younger than 50 years, 638 of 22 631 individuals (2.8%) had an RZV before the expanded indication and 3118 of 23 295 individuals (13.4%) had it by mid-2023. Demographic factors associated with lower odds of vaccination included male sex, African American and unknown race, and nonurban residence (Table 2). Factors associated with increased odds of vaccination included treatment with tsDMARDs and other measures of increased health care use (Table 2). Across VA facilities, the percentage of veterans with 1 or more RZV doses at any time ranged from 17.7% to 71.1%.

**Table 2.** Predictive Margins From GEE Models (N = 190 162)

Variable	Unadjusted predictive margin (95% CI)	*P* value	Adjusted predictive margin (95% CI)^a^	*P* value
Age, y				
<50 (reference)	13.4 (12.9-13.8)	NA	13.5 (12.5-14.6)	NA
≥50	49.8 (49.6-50.1)	<.001	48.8 (47.2-50.4)	<.001
Sex				
Female (reference)	41.7 (41.0-42.3)	NA	47.9 (46.2-49.5)	NA
Male	45.9 (45.6-46.1)	<.001	44.2 (42.7-45.8)	<.001
Self-identified race				
Asian	46.8 (44.6-49.1)	.80	51.7 (48.6-54.8)	<.001
African American	40.9 (40.3-41.4)	<.001	42.1 (40.4-43.7)	<.001
White (reference)	46.6 (46.3-46.8)	NA	45.2 (43.7-46.7)	NA
Other^b^	44.4 (42.7-46.0)	.01	44.3 (41.7-46.9)	.33
Unknown	43.5 (42.7-44.4)	<.001	43.6 (41.9-45.3)	.002
Self-identified ethnicity				
Hispanic (reference)	43.2 (42.2-44.1)	NA	46.5 (44.6-48.4)	NA
Not Hispanic	45.6 (45.3-45.8)	<.001	44.5 (42.9-46.0)	.001
Unknown	43.6 (42.5-44.7)	.53	45.0 (43.2-46.8)	.08
Rurality of residence				
Urban (reference)	45.3 (45.1-45.6)	NA	45.0 (43.5-46.5)	NA
Rural	45.5 (45.1-45.9)	.47	44.1 (42.4-45.7)	.004
Unknown	27.2 (23.7-30.7)	<.001	26.8 (19.5-34.1)	<.001
Visits with >1 specialty since 2018				
No (reference)	42.2 (41.9-42.5)	NA	42.3 (40.8-43.8)	NA
Yes	50.5 (50.1-50.9)	<.001	48.4 (46.7-50.0)	<.001
Medications				
csDMARD monotherapy (reference)	45.1 (44.7-45.5)	NA	43.6 (42.1-45.1)	NA
GC monotherapy	47.1 (46.6-47.6)	<.001	44.1 (42.3-46.0)	.19
Biologic monotherapy	39.4 (38.9-39.9)	<.001	43.6 (42.0-45.1)	.92
Combination of csDMARD, GC, or biologic (no tsDMARDs)	48.2 (47.7-48.7)	<.001	46.5 (44.8-48.1)	<.001
tsDMARD monotherapy or in combination	53.0 (51.9-54.0)	<.001	51.8 (49.7-53.9)	<.001
Other vaccination since 2018				
No (reference)	4.8 (4.4-5.2)	NA	5.6 (4.9-6.4)	NA
Yes	48.1 (47.8-48.3)	<.001	47.0 (45.4-48.6)	<.001

Abbreviations: csDMARD, conventional synthetic disease-modifying antirheumatic drug; GC, glucocorticoid; GEE, generalized estimating equation; NA, not applicable; tsDMARD, targeted synthetic disease-modifying antirheumatic drug.

^a^ Model adjusted for all variables shown in the table.

^b^ Other race includes American Indian or Alaska Native and Native Hawaiian or Other Pacific Islander.

## Discussion

To our knowledge, this cross-sectional study is the first study to examine RZV receipt in a large population of adults, including those younger than 50 years, receiving immunosuppressive medications. Overall, less than half of veterans in the US taking chronic immunosuppressive medications received even 1 dose of RZV; for patients younger than 50 years, percentages were much lower. Although vaccination rates among these older veterans at high risk were higher than those in the 2021 National Health Interview Survey (18.6% for US adults aged ≥50 years),^6^ there remains substantial room for improvement.

This study may not be generalizable to nonveteran populations or non-US countries. Limitations also include difficulty with capture of vaccinations not administered within the VHA system, which may result in an underestimate of the percentage of patients vaccinated. Future work to improve RZV vaccination in patients at high risk should focus on creating informatics tools to identify individuals at high risk and standardizing vaccination guidelines across subspecialties.

## Notes

Supplement 1.
eMethods.

eReferences.

Supplement 2.
Data Sharing Statement
