# Supplementary material for: Recombinant Zoster Vaccination Among US Veterans Receiving Immunosuppressive Medications
Source: JAMA Netw Open. 2024 Oct 11;7(10):e2439945. doi: 10.1001/jamanetworkopen.2024.39945 (PMC11581597; doi:10.1001/jamanetworkopen.2024.39945)
Supplement: Supplement 1. — eMethods. eReferences. [file jamanetwopen-e2439945-s001.pdf]

Supplemental Online Content

Abada S, Li J, Tarasovsky G, et al. Recombinant zoster vaccination among US veterans receiving immunosuppressive medications. *JAMA Netw Open*. 2024;7(10):e2439945. doi:10.1001/jamanetworkopen.2024.39945

eMethods.

eReferences.

This supplemental material has been provided by the authors to give readers additional information about their work.

© 2024 Abada S et al. *JAMA Network Open.*

eMethods

Data source and study design

This cross-sectional study used nationwide VHA Corporate Data Warehouse (CDW) data. The VHA is a national healthcare system serving United States military veterans and is a subdivision within the U.S. Department of Veterans Affairs (VA). The VHA healthcare system is the largest integrated healthcare delivery system in the United States, with more than 9 million living enrollees. This analysis was conducted as part of a quality improvement project funded by the VA's Quality Enhancement Research Initiative (QUERI).

Study population and study period

The study included all patients at 130 medical facilities who were prescribed at least one immunosuppressive medication for at least 90 days between 1/1/2018 and 6/30/2023 by a VA clinician. Immunosuppressive medications of interest were based on the American College of Rheumatology (ACR) 2022 vaccination guidelines, and included conventional synthetic (cs) disease-modifying antirheumatic drugs (DMARDs; azathioprine, cyclophosphamide, cyclosporine, hydroxychloroquine, leflunomide, methotrexate, mycophenolate mofetil, mycophenolic acid, sulfasalazine, tacrolimus, and voclosporin), biologics and any available biosimilars (abatacept, adalimumab, anakinra, anifrolumab, belimumab, certolizumab, canakinumab, etanercept, denosumab, golimumab, guselkumab, infliximab, ixekizumab, obinutuzumab, ocrelizumub, risankizumab, rituximab, rilonacept, sarilumab, secukinumab, tocilizumab, tildrakizumab, and ustekinumab), targeted synthetic (ts) DMARDs (apremilast,

© 2024 Abada S et al. *JAMA Network Open.*

baricitinib, filgotinib, tofacitinib, and upadacitinib), and oral glucocorticoids (GCs; prednisone, cortisone, dexamethasone, hydrocortisone, methylprednisolone, and prednisolone) at dose of ≥10 mg daily prednisone equivalents.[1] This glucocorticoid dose threshold was chosen based on existing literature which has identified a consistently increased risk of HZ with ≥10 mg daily prednisone equivalents.[2–6] Patients age <18 years were excluded.

Outcomes

RZV documentation data was extracted from both vaccination tables and "health factors" which are structured fields available at each facility that capture additional RZV records (e.g., "Shingrix outside site").[7] RZV was defined using CVX codes 187 ("zoster recombinant") during the study period or 188 ("zoster vaccine, unspecified formulation") documented after 1/1/2021 (when the live-attenuated zoster vaccine was no longer available in the United States). CVX codes indicate the product used in a vaccination and are maintained by the Centers for Disease Control and Prevention. The outcome of interest was the percentage of veterans in the study population who had any RZV (at least 1 dose). The percentage with any RZV before the expanded indication (1/1/2018 – 2/28/2022) was calculated as the number of veterans who had any RZV documented during that time divided by the number of veterans who were prescribed ≥ 1 immunosuppressive medication for over 90 days during the same period AND ≥ 50 years old by 8/31/2021. We required veterans to be at least 50 years old 6 months prior to the end of the time window in order to allow time for them to have an encounter during which they could be vaccinated. The percentage with any RZV documented over the entire study period was calculated similarly but without the age restriction: the number of veterans with any

© 2024 Abada S et al. *JAMA Network Open.*

RZV documented during the entire study period (1/1/2018 – 6/30/2023) divided by the number of veterans with ≥ 1 immunosuppressive medication for over 90 days during the same period.

We aggregated individual patient data to the facility level, expressed as the percentage of veterans in the study population at that facility who had at least 1 dose of RZV documented at any time. Since patients who received the live-attenuated vaccine are still recommended to receive two doses of RZV, live-attenuated vaccination status was not included in the study analysis.

Patient characteristics

Patient characteristics included in this study were age, gender, self-identified race (African American, Asian, White, other, and unknown) and self-identified ethnicity (Hispanic, not Hispanic, and unknown) as recorded in the EHR, Veteran rurality based on residential address (urban, rural, or unknown), visits to specific VHA specialty clinics during the study period (Rheumatology, Gastroenterology, Neurology, Oncology; more than one specialty clinic visit, and none), other vaccinations received at the VHA during the study period (including influenza, pneumococcal, etc.; yes/no), and rheumatic condition (rheumatoid arthritis, other inflammatory arthritis, spondylitis, systemic lupus erythematosus, sarcoidosis, ANCA-associated vasculitis, or mixed connective tissue disease (MTCD)) based on the presence of at least one ICD-10 code for these conditions.[8] "Other" race included individuals self-identified as American Indian or Alaska Native, as well as individuals self-identified as Native Hawaiian or Other Pacific Islander. Self-identified race and ethnicity were assessed in this study to identify possible

disparities in RZV receipt among self-identified groups; knowledge of such disparities could help identify specific subgroup needs and assist with the design of interventions for those subgroups. Rheumatic conditions were not mutually exclusive.

Facility characteristics

Facility-level covariates included geographical region (Midwest, North Atlantic, Continental, Southeast, Pacific) and complexity (High, Medium, Low). Facility complexity was assigned by the VHA based on a variety of factors (morbidity of patient population, presence of clinical services such as intensive care unit and surgical operating rooms, administrative complexity, and presence of graduate medical education programs.[9]

Statistical analysis

Descriptive statistics were used to examine patient and practice characteristics according to the RZV documentation status. Bivariate analyses were completed by chi-square tests for categorical variables and t-tests for continuous variables.

To examine factors associated with RZV documentation at any time, we constructed a multi-level regression model that included age, gender, race, ethnicity, rurality of residence, visits with >1 subspecialty during the study period (yes/no), receipt of at least one other vaccination at the VA during the study period (yes/no), and medication type (csDMARD monotherapy, GCs monotherapy, combinations of csDMARD – GCs – biologics (other combination without

© 2024 Abada S et al. *JAMA Network Open.*

tsDMARDs), and tsDMARD monotherapy or in combination (any tsDMARDs)), accounting for clustering by practice using generalized estimating equations (GEE). Multicollinearity was examined using the variance inflation factor.

Facility-level performance at any time was reported as the percentage of patients with at least one dose of RZV documented among all those eligible within a given facility using median and interquartile ranges. Facilities with fewer than 20 patients were excluded from the facility-level analysis. For all models, model output parameters were used to produce predicted probabilities and 95% confidence intervals. A 2-sided *P* value <.05 was considered statistically significant. Analyses were performed using Stata 16 (StataCorp. 2017. College Station, TX: StataCorp LLC).

This work was approved by the VA Quality Enhancement Research Initiative (QUERI; IRB 15-18358). We followed the Strengthening the Reporting of Observational Studies in Epidemiology (STROBE) reporting guidelines for cross-sectional studies (see Supplement 1).

© 2024 Abada S et al. *JAMA Network Open.*

eReferences

1. *Project Plan - April 2021: Guideline for Vaccinations in Patients with Rheumatic and Musculoskeletal Diseases*. American College of Rheumatology. Accessed February 20, 2024. https://assets.contentstack.io/v3/assets/bltee37abb6b278ab2c/bltcf56d55226298e0b/6331c8f61885443554793107/vaccinations-guideline-project-plan-2022.pdf

2. Yun H, Yang S, Chen L, et al. Risk of Herpes Zoster in Autoimmune and Inflammatory Diseases: Implications for Vaccination. *Arthritis & Rheumatology*. 2016;68(9):2328-2337. doi:10.1002/art.39670

3. Yun H, Xie F, Delzell E, et al. Risks of Herpes Zoster in Patients With Rheumatoid Arthritis According to Biologic Disease-Modifying Therapy. *Arthritis Care & Research*. 2015;67(5):731-736. doi:10.1002/acr.22470

4. Pappas DA, Hooper MM, Kremer JM, et al. Herpes Zoster Reactivation in Patients With Rheumatoid Arthritis: Analysis of Disease Characteristics and Disease-Modifying Antirheumatic Drugs. *Arthritis Care & Research*. 2015;67(12):1671-1678. doi:10.1002/acr.22628

5. Winthrop KL, Baddley JW, Chen L, et al. Association Between the Initiation of Anti–Tumor Necrosis Factor Therapy and the Risk of Herpes Zoster. *JAMA*. 2013;309(9):887. doi:10.1001/jama.2013.1099

© 2024 Abada S et al. *JAMA Network Open.*

6. Yang SC, Lai YY, Huang MC, Tsai CS, Wang JL. Corticosteroid dose and the risk of opportunistic infection in a national systemic lupus erythematosus cohort. *Lupus*. 2018;27(11):1819-1827. doi:10.1177/0961203318792352

7. Melzer AC, Pinsker EA, Clothier B, et al. Validating the use of veterans affairs tobacco health factors for assessing change in smoking status: accuracy, availability, and approach. *BMC Med Res Methodol*. 2018;18(1):39. doi:10.1186/s12874-018-0501-2

8. Schmajuk G, Montgomery AD, Leonard S, et al. Factors Associated With Hospitalization and Death After COVID-19 Diagnosis Among Patients With Rheumatic Disease: An Analysis of Veterans Affairs Data. *ACR Open Rheumatology*. 2021;3(11):796-803. doi:10.1002/acr2.11328

9. *Site Facility Name and Complexity*. United States Department of Veterans Affairs. Accessed January 20, 2022. https://www.vendorportal.ecms.va.gov/FBODocumentServer/DocumentServer.aspx?DocumentId=2793591&FileName=VA118-16-R-1059-A00002002.docx

© 2024 Abada S et al. *JAMA Network Open.*
