# Supplementary material for: Recombinant Zoster Vaccination Among US Veterans Receiving Immunosuppressive Medications
Source: JAMA Netw Open. 2024 Oct 11;7(10):e2439945. doi: 10.1001/jamanetworkopen.2024.39945 (PMC11581597; doi:10.1001/jamanetworkopen.2024.39945)
Supplement: Supplement 2. — Data Sharing Statement [file jamanetwopen-e2439945-s002.pdf]

Data Sharing Statement

Abada. Recombinant Zoster Vaccination Among US Veterans Receiving Immunosuppressive Medications. *JAMA Netw Open*. Published online October 11, 2024. doi:10.1001/jamanetworkopen.2024.39945

Data
Data available: No

Additional Information
Explanation for why data not available: VHA data is only available to approved VHA investigators.
